# A reflection on lithium-ion battery cathode chemistry

**DOI:** 10.1038/s41467-020-15355-0

**Published:** 2020-03-25

**Authors:** Arumugam Manthiram

**Affiliations:** Materials Science and Engineering Program & Texas Materials Institute, University of Texas at Austin, Austin, TX 78712 USA

**Keywords:** Solid-state chemistry, Batteries, Batteries

## Abstract

Lithium-ion batteries have aided the portable electronics revolution for nearly three decades. They are now enabling vehicle electrification and beginning to enter the utility industry. The emergence and dominance of lithium-ion batteries are due to their higher energy density compared to other rechargeable battery systems, enabled by the design and development of high-energy density electrode materials. Basic science research, involving solid-state chemistry and physics, has been at the center of this endeavor, particularly during the 1970s and 1980s. With the award of the 2019 Nobel Prize in Chemistry to the development of lithium-ion batteries, it is enlightening to look back at the evolution of the cathode chemistry that made the modern lithium-ion technology feasible. This review article provides a reflection on how fundamental studies have facilitated the discovery, optimization, and rational design of three major categories of oxide cathodes for lithium-ion batteries, and a personal perspective on the future of this important area.

## Abstract (web summary)

The 2019 Nobel Prize in Chemistry has been awarded to a trio of pioneers of the modern lithium-ion battery. Here, Professor Arumugam Manthiram looks back at the evolution of cathode chemistry, discussing the three major categories of oxide cathode materials with an emphasis on the fundamental solid-state chemistry that has enabled these advances.

## Introduction

Lithium-ion batteries have become an integral part of our daily life, powering the cellphones and laptops that have revolutionized the modern society^1–3^. They are now on the verge of transforming the transportation sector with electric cars, buses, and bikes. They are also anticipated to be critical for enabling a widespread replacement of fossil-fuel-based power generation with renewable energy sources like solar and wind, providing a cleaner, more sustainable planet. The award of the 2019 Nobel Prize in Chemistry to John Goodenough, Stanley Whittingham, and Akira Yoshino emboldens this assertion.

The development of lithium-ion battery technology to date is the result of a concerted effort on basic solid-state chemistry of materials for nearly half a century now. Discovery of new materials and a deepening of our fundamental understanding of their structure-composition-property-performance relationships have played a major role in advancing the field. Among the various components involved in a lithium-ion cell, the cathodes (positive electrodes) currently limit the energy density and dominate the battery cost. It is interesting to realize that all the three leading oxide cathode chemistries (layered, spinel, and polyanion families) currently in use originated from John Goodenough’s group at the University of Oxford in England and at the University of Texas at Austin (UT Austin) in the United States. It is timely to take a deep look and reflect on the evolution of lithium-ion battery cathode chemistry, which is the purpose of this review article. The article will serve as an embodiment of how collective contributions of young and experienced minds can work together to deliver wonders in science and technology, inspiring new generations to make discoveries through basic science research.

## The birth of rechargeable lithium batteries

Intercalation chemistry involving reactions between guest molecules or ions with solid hosts has been known for nearly 180 years^4^. Schauffautl was the first to show the intercalation of sulfate ions into graphite in 1841. However, the interest in intercalation materials became prominent only in the 1960s, particularly with respect to altering the electronic and optical properties of materials through guest ion intercalation^5–7^. A few transition-metal disulfides MS_2_ as well as oxides such as WO_3_ were investigated by intercalating A = H^+^, Li^+^, and Na^+^ ions^4^. For instance, the intercalation of these monovalent ions into WO_3_ to produce A_*x*_WO_3_ altered the electronic conductivity from insulator to semiconductor to metallic depending on the value of *x*. These intercalation reactions were also accompanied with structural changes with rich crystal chemistry.

With the chemical intercalation reactions on metal disulfides in place, Whittingham^8^ demonstrated the first rechargeable lithium battery at Exxon Corporation in the United States with a TiS_2_ cathode, a lithium-metal anode, and a liquid electrolyte in which a lithium salt like LiClO_4_ was dissolved in an organic solvent like dimethoxyethane (glyme) and tetrahydrofuran (THF). The Li-TiS_2_ cell displayed a discharge voltage of <2.5 V with good reversibility for one lithium per TiS_2_ molecule. Following the demonstration with TiS_2_, a number of metal dichalcogenides were investigated by various groups as electrode materials for lithium batteries^4^. However, there were two major issues. First, the cell voltage was limited to <2.5 V, limiting the energy density. Second, dendrite growth on lithium-metal anodes during cell cycling caused internal shorting and presented a fire hazard. In fact, there were attempts to put cells consisting of sulfide cathodes and lithium-metal anodes into market, but they were then abandoned due to safety issues^9,10^.

## The discovery of oxide cathodes

**Fig. 1:**
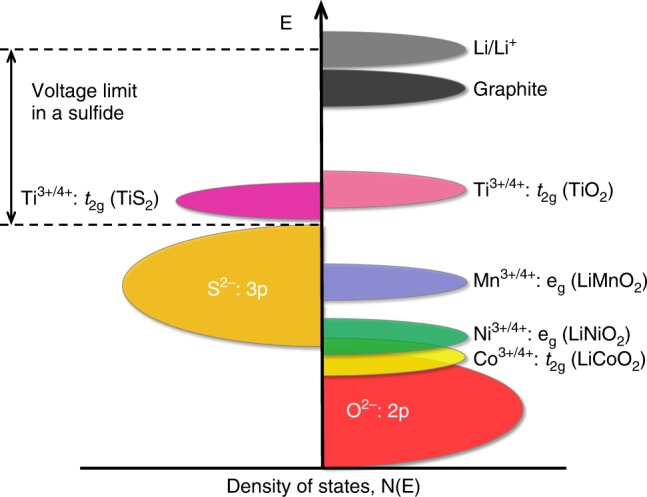
Positions of the redox energies relative to the top of the anion: p bands. The top of the S^2−^:3p band lying at a higher energy limits the cell voltage to <2.5 V with a sulfide cathode. In contrast, the top of the O^2−^:2p band lying at a lower energy enables access to lower-lying energy bands with higher oxidation states and increases the cell voltage substantially to ~4 V.

With an aim to increase the cell voltage and to develop cathodes with lithium already in them, Goodenough’s group began to explore oxide cathodes in the 1980s at the University of Oxford in England. The cell voltage is determined by the energy difference between the redox energies of the anode and the cathode. This means that the cathode energy should lie as low as possible and the anode energy should lie as high as possible, which implies that the cathode would require the stabilization of higher oxidation states with a lower-lying energy band while the anode would require the stabilization of lower oxidation states with a higher-lying energy band. Therefore, the question is how to access the lower-lying energy band of a metal ion with high enough oxidation states in a material so that the cell voltage can be increased. After three decades of fundamental research between 1950 and 1980 on the properties of materials, particularly transition-metal oxides^11^, Goodenough utilized the basic understanding that the top of the S^2–^:3p band lies at a higher energy than the top of the O^2–^:2p band to design oxide cathodes (Fig. 1). This means that the access to lower-lying energy bands with higher oxidation states such as Co^3+/4+^ and hence the higher cell voltage will be limited by the top of the S^2–^:3p band, and attempts to lower the cathode redox energy by accessing higher oxidation states in a sulfide will result in an oxidation of S^2–^ ions to molecular disulfide ions (S_2_)^2–^. In contrast, in an oxide, the cathode redox energy can be significantly lowered by accessing lower-lying energy bands such as Co^3+/4+^ and hence the cell voltage can be increased to as high as 4 V as the top of the O^2–^:2p band lies at a lower energy compared to the top of the S^2–^:3p band.

**Fig. 2:**
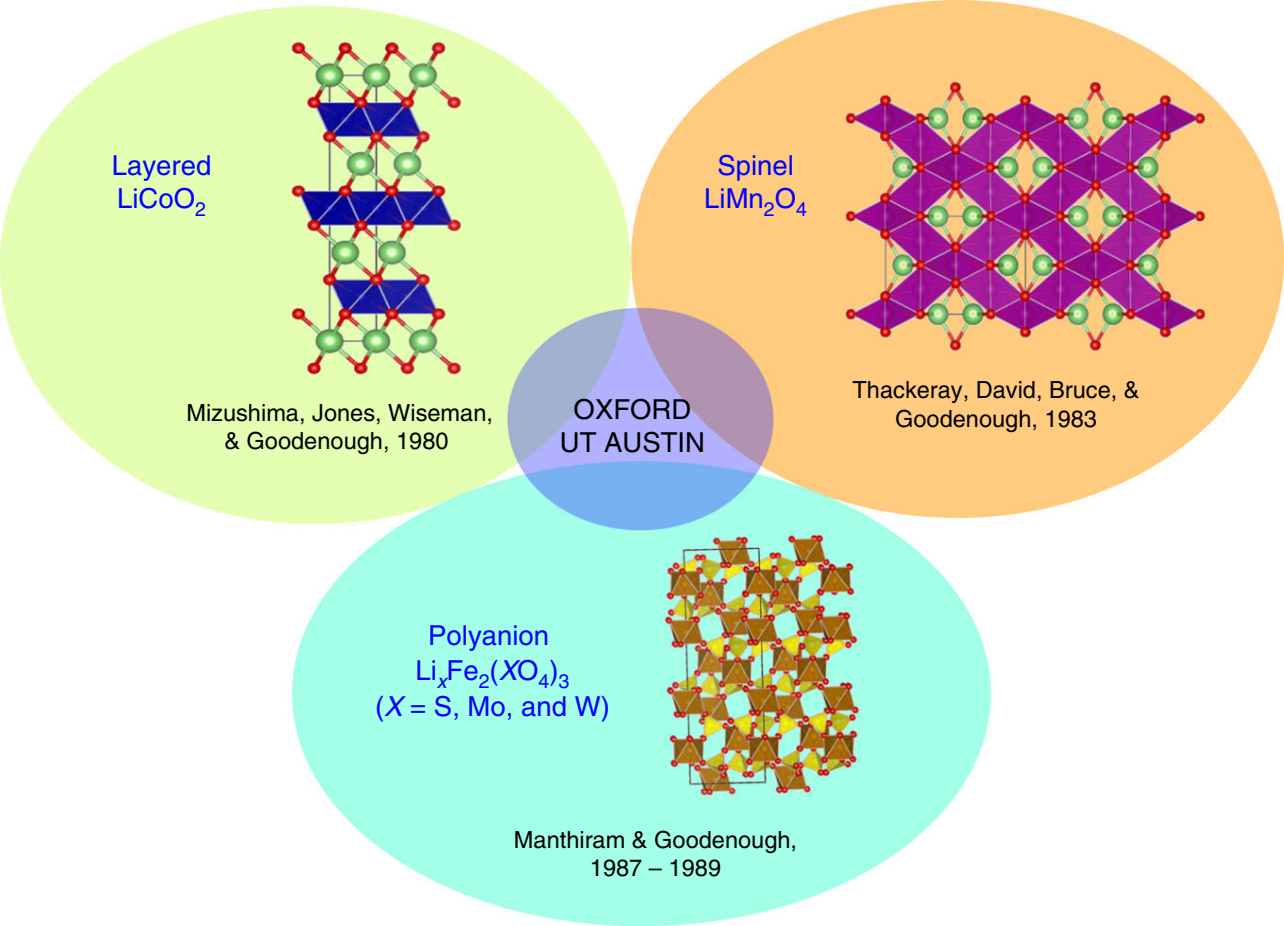
Discovery of three classes of oxide cathodes in the 1980s. Layered LiCoO_2_ with octahedral-site lithium ions offered an increase in the cell voltage from <2.5 V in TiS_2_ to ~4 V. Spinel LiMn_2_O_4_ with tetrahedral-site lithium ions offered an increase in cell voltage from 3 V for octahedral-site lithium ions with Mn^3+/4+^ couple to ~4 V, with an accompanying cost reduction. Polyanion oxide Li_*x*_Fe_2_(SO_4_)_3_ offered yet another way to increase the cell voltage through inductive effect from <2.5 V in a simple oxide like Fe_2_O_3_ to 3.6 V, with a further reduction in cost and improved thermal stability and safety. Oxford and UT Austin, refer, respectively, to the University of Oxford and the University of Texas at Austin.

This basic idea led to the discovery of three classes of oxide cathodes by Goodenough’s group in the 1980s, involving three visiting scientists from three different parts of the world, including Koichi Mizushima from Japan who worked on the layered oxide cathodes, Michael Thackeray from South Africa who worked on the spinel oxide cathodes, and Arumugam Manthiram from India who worked on the polyanion cathodes (Fig. 2). The three visiting scientists began to work with Goodenough in the 1980s and became part of a larger endeavor that has impacted the society enormously. The three of them had no overlap in Goodenough’s group. Mizushima came to work on layered oxide cathodes and left, Thackeray came to work on spinel oxide cathodes and left, and Manthiram came to work on polyanion oxide cathodes, but continued from the University of Oxford to the University of Texas at Austin. The sections below will briefly discuss the discovery of the three classes of oxide cathodes in the 1980s from a solid-state chemistry and physics perspective, which remain as the sole practical cathode classes for lithium-ion batteries. The layered and polyanion classes also serve as the basis for sodium-ion battery cathodes.

## Cathode class I: layered oxides

**Fig. 3:**
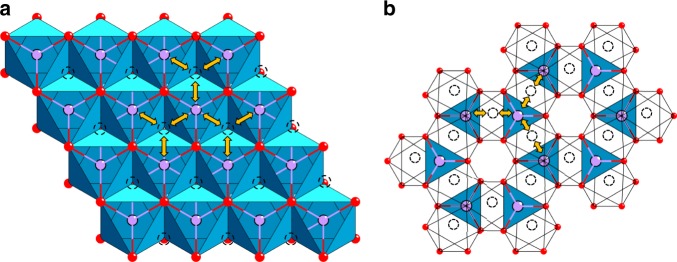
Lithium-diffusion pathways with lower energy barriers in close-packed oxides. **a** Two-dimensional lithium diffusion from one octahedral site to another octahedral site in the lithium plane through a neighboring empty tetrahedral site in the O3 layered LiMO_2_ cathodes. **b** Three-dimensional lithium diffusion from one 8a tetrahedral site to another 8a tetrahedral site through a neighboring empty 16c octahedral site in the spinel cathodes.

The first oxide cathode investigated is the layered LiCoO_2_ (Fig. 2), in which the monovalent Li^+^ and trivalent Co^3+^ ions are ordered on the alternate (111) planes of the rock salt structure with a cubic close-packed array of oxide ions^12^: this structure is referred to as the O3 structure. The large charge and size differences between Li^+^ and Co^3+^ ions lead to good cation ordering, which is critical to support fast two-dimensional lithium-ion diffusion and conductivity in the lithium plane. The lithium-ion conduction in the lithium plane occurs from one octahedral site to another via a neighboring tetrahedral void that shares faces with three octahedra within the lithium layer as it offers the lowest energy barrier (Fig. 3a). With a good cation ordering, the direct Co-Co interaction across the shared octahedral edges in the cobalt plane facilitates good electronic conductivity as well; in fact, Li_1–*x*_CoO_2_ becomes metallic on charging due to the introduction of holes into the low-spin Co^3+/4+^: t_2g_^6–*x*^ band^13,14^. The good structural stability along with high electrical and lithium-ion conductivity offers fast charge–discharge characteristics with good reversibility. With these features, LiCoO_2_ remains as one of the best cathodes to date with a high operating voltage of ~4 V. The LiCoO_2_ cathode solved two major challenges associated with the sulfide cathodes pursued in the 1970s. It enabled not only a substantial increase in the operating voltage from <2.5 V to ~4 V but also the assembly of a cell without the need to employ a metallic lithium anode. As the as-synthesized cathode contained lithium, a lithium-free anode like graphite can be paired with LiCoO_2_ to give the modern-day lithium-ion cell. However, the Co^3+/4+^ band overlaps with the top of the O^2–^:2p band as seen in Fig. 1, which leads to a release of oxygen from the crystal lattice on charging more than 50% with (1 – *x*) < 0.5 in the Li_1–*x*_CoO_2_ cathode^15,16^. Therefore, despite good electrochemical performance, the practical capacity of LiCoO_2_ is limited to ~140 mA h g^–1^.

Following LiCoO_2_, a number of layered LiMO_2_ oxides have been investigated over the years where M = 3d transition metals (Table 1). Some of them can be directly synthesized by direct high-temperature reactions as indicated by “yes” in Table 1. Although both LiMnO_2_ and LiFeO_2_ do not crystallize in the O3 structure when synthesized by high-temperature reactions, they could be obtained by ion-exchanging the sodium analogues (NaMO_2_) with lithium salts^17^. Among them, the M = V, Mn, and Fe members suffer from layered to spinel transitions or other structural changes during charge–discharge^17,18^ due to a low octahedral-site stabilization energy (OSSE) (Table 2), so they are not good cathodes. LiTiO_2_ operates at a lower voltage of ~1.5 V, so it is not suitable as a cathode (Fig. 1). In addition, it is rather tedious to synthesize it with lower-valent Ti^3+^. LiCrO_2_ is difficult to charge as it displays a large polarization with an increase in charge voltage. LiNiO_2_ is also difficult to synthesize as a well-ordered material, unlike LiCoO_2_, as Ni^3+^ tends to be reduced to Ni^2+^ and result in Li_1–*y*_Ni_1+*y*_O_2_ at the high-temperature synthesis conditions of 700–800 °C, accompanied by a volatilization of some lithium from the reaction mixture^19,20^.

**Table 1:** LiMO_2_ oxides crystallizing in the O3 layered structure of LiCoO_2_.

M^3+^ ion	Sc	Ti	V	Cr	Mn	Fe	Co	Ni	Cu
LiMO_2_	No	Yes	Yes	Yes	No	No	Yes	Yes	No

**Table 2:** Comparison of the crystal field stabilization energies (CFSE) of the transition-metal ions M^3+^ in NMC cathodes.

Ion	Octahedral CFSE^a^	Tetrahedral CFSE^b^	Octahedral-site stabilization energy (OSSE)^c^
Mn^3+^: d^4^	t_2_^3^e^1^: −0.6 Δ_o_	e^2^t_2_^2^: −0.18 Δ_o_	−0.42 Δ_o_
Ni^3+^: d^7^	t_2_^6^e^1^: −1.88 Δ_o_	e^4^t_2_^3^: −0.53 Δ_o_	−1.35 Δ_o_
Co^3+^: d^6^	t_2_^6^e^0^: −2.4 Δ_o_	e^3^t_2_^3^: −0.27 Δ_o_	−2.13 Δ_o_

^a^Δ_o_ Refers to octahedral-site splitting; for simplicity, the pairing energies are ignored.

^b^tetrahedral splitting Δ_t_ was converted to octahedral splitting by the relation Δ_t_ = 0.44 Δ_o._

^c^OSSE = octahedral CFSE − tetrahedral CFSE.

The high cost and limited capacity of LiCoO_2_ have, however, been driving the substitution of cobalt with Mn and Ni during the past couple of decades to give LiNi_1–*y*–*z*_Mn_*y*_Co_*z*_O_2_ (NMC). The question is why use NMC with three transition-metal ions. In NMC, Mn^3+^ tends to get oxidized during synthesis to Mn^4+^ by reducing Ni^3+^ to Ni^2+^ as the Mn^3+/4+^ band lies above the Ni^2+/3+^ band. Thus, Mn^4+^ helps the incorporation of Ni as a stable Ni^2+^ into NMC and serves as a structural stabilizer without participating in the charge–discharge process. In NMC, each transition-metal ion has its own advantages and disadvantages (Table 3). The two major factors are chemical stability and structural stability, in which Co and Mn are diametrically opposite to each other. Since the Mn^3+/4+^ band lies well above the top of the O^2–^:2p band (Fig. 1), Mn does not suffer from any chemical instability involving oxygen release from the lattice in contrast to Co as the Co^3+/4+^ band overlaps with the top of the O^2–^:2p band. On the other hand, Mn suffers from structural instability as it can readily migrate from the octahedral sites in the transition-metal plane to the octahedral sites in the lithium plane through a neighboring tetrahedral site due to the smaller OSSE as seen in Table 2, resulting in a layered to spinel transition and accompanying voltage decay during cycling. In contrast, Co enjoys good structural stability without such cation migration due to the large OSSE. In addition to the above two critical differences, Co^3+/4+^ becomes metallic on charging due to the partially filled t_2g_ orbitals interacting along the shared edges^13,14^, while Mn^4+^ remains semiconducting. Also, Mn is abundant and environmentally benign compared to Co. Interestingly, Ni lies in between Mn and Co in all the five criteria in Table 3 as (i) the Ni^3+/4+^ band barely touches the top of the O^2–^:2p band so that Ni^3+^ can be charged all the way to Ni^4+^ without the removal of electron density from the O^2–^:2p band and loss of oxygen from the lattice and (ii) Ni^3+^ exhibits an OSSE value intermediate between those of Mn^3+^ and Co^3+^, offering reasonably good structural stability. That is why the trend is to progressively increase the Ni content and decrease the Co content in NMC so that the capacity can be increased while lowering the cost. This will be discussed further below.

**Table 3:** Comparison of the characteristics of Mn, Co, and Ni in NMC cathodes.

Parameter	Trend
Chemical stability	Mn > Ni > Co
Structural stability	Co > Ni > Mn
Electrical conductivity	Co > Ni > Mn
Abundance	Mn > Ni > Co
Environmental benignity	Mn > Ni > Co

## Cathode class II: spinel oxides

With a prior demonstration of lithium insertion into magnetite (Fe_3_O_4_) crystallizing in the spinel structure by Thackeray in South Africa^21^, the second class of cathode discovered is the spinel LiMn_2_O_4_ at the University of Oxford (Fig. 2), in which the Mn^3+/4+^ ions occupy the 16d octahedral sites and the Li^+^ ions occupy the 8a tetrahedral sites of the spinel framework with a cubic close-packed array of oxide-ions^22^. The stable [Mn_2_]_16d_O_4_ framework with edge-shared octahedra offers a three-dimensional lithium-ion diffusion pathway with fast lithium-ion conductivity. The lithium-ion conduction occurs from one 8a tetrahedral site to another 8a tetrahedral site via a neighboring empty 16c octahedral site as it offers the lowest energy barrier (Fig. 3b). The direct Mn–Mn interaction across the shared MnO_6_ octahedral edges as in LiCoO_2_ with mixed-valent high-spin Mn^3+/4+^:t_2g_^3^e_g_^1–*x*^ in (Li_1–*x*_)_8a_[Mn_2_]_16d_O_4_ facilitates good hopping electronic conduction, but it remains as a small-polaron semiconductor during the charge–discharge process, unlike Li_1–*x*_CoO_2_. The good three-dimensional structural stability along with high electrical and lithium-ion conductivity offers even faster charge–discharge characteristics for Li_1–*x*_Mn_2_O_4_ with good reversibility compared to LiCoO_2_. The insertion/extraction of lithium into/from the tetrahedral sites with a deep site energy in Li_1–*x*_Mn_2_O_4_ offers a high operating voltage of 4 V with a practical capacity of <130 mA h g^–1^ as close to one lithium per two Mn ions can be reversibly extracted from the tetrahedral sites.

Interestingly, an additional lithium can be inserted into the empty 16c octahedral sites at 3 V to form the lithiated spinel [Li_2_]_16c_[Mn_2_]_16d_O_4_ that is accompanied by a spontaneous displacement of the already existing 8a tetrahedral-site lithium ions into the empty 16c octahedral sites. It is interesting to note that the operating voltage drops by 1 V on going from a tetrahedral-site to octahedral-site lithium, despite the same Mn^3+/4+^ redox couple. This illustrates the significant role of site energy in addition to electron transfer in controlling the voltage in solids, unlike in solutions where no site energy contribution is involved. However, the accompanying Jahn-Teller distortion caused by a single e_g_ electron in Mn^3+^:t_2g_^3^e_g_^1^ in [Li_2_]_16c_[Mn_2_]_16d_O_4_ causes a cubic to tetragonal phase transition with a two-phase reaction involving a large instantaneous *c/a* ratio and volume changes^23^. Therefore, the capacity in the 3 V region could not be utilized in practical cells.

One important advantage on going from LiCoO_2_ to LiMn_2_O_4_ is the significant reduction in cost as Mn is two orders of magnitude lower in cost than Co. However, one critical issue with LiMn_2_O_4_ is the dissolution of Mn from the lattice into the electrolyte in presence of trace amounts (ppm levels) of H^+^ ions (acidity) in the electrolyte^24,25^ due to the well-known disproportionation of Mn^3+^ to Mn^4+^ and Mn^2+^ in acid^26^. During such a disproportionation, Mn^4+^ is retained in the solid and Mn^2+^ is leached out into the solution. In addition to degrading the cathode, the Mn dissolution and its migration to the anode severely poison the graphite anode and limit the cycle life of lithium-ion cells^27^. Interestingly, substituting a small amount of lithium (e.g., 5 atom %) for Mn in LiMn_2_O_4_ perturbs the long-range Mn–Mn interaction/contact, frustrates the Mn^3+^ disproportionation reaction, reduces Mn dissolution, and thereby improves the cyclability.

Unfortunately, LiM_2_O_4_ spinel oxides are known only with M = Ti, V, and Mn, unlike the layered LiMO_2_ oxides (Table 4). This is because of the difficulty of stabilizing the highly oxidized M^3+/4+^ oxidation states by conventional high-temperature synthesis. Among the known three spinel oxides, LiTi_2_O_4_ operates around 1.5 V, so it is not a suitable cathode. Nor is LiV_2_O_4_ a practical choice since it suffers from structural changes and a lower voltage of around 3 V^28^. There have been efforts to prepare both LiCo_2_O_4_ and LiNi_2_O_4_ spinel oxides by chemically extracting 50% lithium, respectively, from LiCoO_2_ and LiNiO_2_ to obtain Li_0.5_CoO_2_ and Li_0.5_NiO_2_, followed by calcining them at moderate temperatures of 200–400 ^o^C to transform the layered phase to spinel phase^19,29–31^. However, such attempts result in either incomplete transformation at low enough temperatures or loss of oxygen and formation of a mixture of spinel-like phases and reduced Co_3_O_4_ or NiO phases due to the instability of Co^3+/4+^ and Ni^3+/4+^ at high enough temperatures. Such spinel-like phases also exhibit poor electrochemical performance due to a lack of well-formed crystalline spinel phases. Another approach has been to partially substitute Mn with other ions like Cr, Co, and Ni. One example is LiMn_1.5_Ni_0.5_O_4_ spinel^32,33^, in which Mn exists as Mn^4+^ and Ni exists as Ni^2+^ as in NMC cathodes. With Ni^2+/3+^ and Ni^3+/4+^ couples and tetrahedral-site lithium ions, LiMn_1.5_Ni_0.5_O_4_ operates at ~4.7 V with a reversible capacity of ~135 mA h g^–1^. However, LiMn_1.5_Ni_0.5_O_4_ spinel suffers from capacity fade due to the lack of suitable electrolyte that can be stable at such high voltages.

**Table 4:** LiM_2_O_4_ oxides crystallizing in the spinel structure.

M^3+/4+^	Sc	Ti	V	Cr	Mn	Fe	Co	Ni	Cu
LiM_2_O_4_	No	Yes	Yes	No	Yes	No	No	No	No

## Cathode class III: polyanion oxides

Departing from the previous two simple oxide classes of cathodes, the third class of cathode investigated is the polyanion oxides. Based on Manthiram’s Ph.D. dissertation work in India on the hydrogen reduction of the polyanion oxides Ln_2_(MoO_4_)_3_ (Ln = lanthanide and Y) to obtain lower-valent Mo^4+^ oxides^34^ Ln_2_(MoO_3_)_3_, analogous polyanion oxides Fe_2_(MoO_4_)_3_ and Fe_2_(WO_4_)_3_ were prepared by Manthiram, crystallizing in a NASICON-related framework structure (Fig. 2). These polyanion oxides were found to undergo reversible insertion/extraction of two lithium ions per formula unit to give Li_2_Fe_2_(MoO_4_)_3_ or Li_2_Fe_2_(WO_4_)_3_ both by chemical and electrochemical methods^35^. Interestingly, both Fe_2_(MoO_4_)_3_ and Fe_2_(WO_4_)_3_ exhibited a flat discharge voltage of 3 V, which was significantly higher than that seen with simple oxides like Fe_2_O_3_ or Fe_3_O_4_ (<2.5 V) operating with the same Fe^2+/3+^ redox couple^21^. Motivated by the increase in voltage on going from a simple oxide to a polyanion oxide, Fe_2_(SO_4_)_3_ having the same structure as Fe_2_(MoO_4_)_3_ was then investigated^36^. Surprisingly, Fe_2_(SO_4_)_3_ displayed a much higher flat discharge voltage of 3.6 V.

**Fig. 4:**
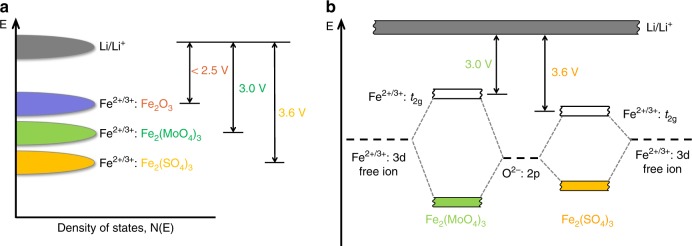
Role of counter-cations in shifting the redox energies in polyanion oxides. **a** Lowering of the redox energies of the Fe^2+/3+^ couple and the consequent increase in cell voltage on going from a simple oxide Fe_2_O_3_ to a polyanion oxide Fe_2_(MoO_4_)_3_ and then to another polyanion oxide Fe_2_(SO_4_)_3_ with a more electronegative counter-cation S^6+^ vs. Mo^6+^, i.e., with a more covalent S–O bond than the Mo–O bond. **b** Molecular orbital energy diagram illustrating the lowering of the Fe^2+/3+^ redox energy in Fe_2_(SO_4_)_3_ compared to that in the isostructural Fe_2_(MoO_4_)_3_, due to a weakening of the Fe–O covalence by a more covalent S–O bond than the Mo–O bond through inductive effect.

A comparison of the operating voltages of the isostructural Fe_2_(MoO_4_)_3_ (3.0 V), Fe_2_(WO_4_)_3_ (3.0 V), and Fe_2_(SO_4_)_3_ (3.6 V) with that of Fe_2_O_3_ (<2.5 V), all operating with the same Fe^2+/3+^ couple (Fig. 4), led to a recognition of the role of the counter cations Mo^6+^, W^6+^, and S^6+^ in drastically shifting the redox energy of the Fe^2+/3+^ couple by altering the characteristics of the Fe–O bond. In the Fe_2_(XO_4_)_3_ (X = Mo, W, and S) structure (Fig. 2), the FeO_6_ octahedra share their corners with XO_4_ tetrahedra, providing an extended, three-dimensional –O–Fe–O–X–O–Fe–O– linkage. As a result, the more covalent Mo–O or W–O bond weakens the Fe–O bond covalency through inductive effect compared to that in the simple iron oxide Fe_2_O_3_, resulting in a lowering of the Fe^2+/3+^ redox energy and a consequent increase in the operating voltage from <2.5 to 3.0 V in Fe_2_(MoO_4_)_3_ and Fe_2_(WO_4_)_3_. An even more covalent S-O bond in Fe_2_(SO_4_)_3_ weakens the Fe–O covalency ever further, resulting in a further lowering of the Fe^2+/3+^ redox energy and a much more significant increase in the operating voltage from 3.0 V in Fe_2_(MoO_4_)_3_ and Fe_2_(WO_4_)_3_ to 3.6 V in Fe_2_(SO_4_)_3_ (Fig. 4).

Intrigued by the ability to increase the operating voltage by going from simple oxides to polyanion oxides in addition to the increase in voltage from a sulfide to an oxide in the previous two classes of cathodes, polyanion oxides with phosphate groups were investigated by Manthiram and Goodenough in the late 1980s. In the meantime, high-temperature copper oxide superconductors came along in the late 1980s, so Manthiram and Goodenough became preoccupied with these exciting superconducting oxides^37^. Therefore, the phosphate project was proposed by Manthiram and Goodenough to a Ph.D. student Geeta Ahuja who continued exploring them. Thus, the investigation of polyanion phosphates, such as LiTi_2_(PO_4_)_3_, LiZrTi(PO_4_)_3_, NbTi(PO_4_)_3_, and SbTi(PO_4_)_3_ became part of the Ph.D. dissertation of Geeta Ahuja^38^ in 1991. Ahuja’s Ph.D. dissertation work on the above four materials focused largely on the analysis by x-ray diffraction of the phases formed and delineating single-phase vs. two-phase reactions during chemical lithiation. The work also found that all of them had an operating voltage of 2–3 V, with the Li_*x*_SbTi(PO_4_)_3_ system displaying the highest voltage of 3 V, illustrating that the inductive effect by the phosphate group significantly increases the voltage to 2–3 V compared to ~1.5 V for the Ti^3+/4+^ couple in a simple oxide like TiO_2_. Unfortunately, the results of these investigations were not published as a journal article, as these materials were not considered appealing at the time either as a cathode or as an anode due to an intermediate operating voltage of 2–3 V along with the undesired two-phase reactions and poor electronic conductivity. In the meantime, Sony Corporation announced in 1991 the commercialization of lithium-ion batteries with LiCoO_2_ cathode and graphite anode. Motivated by this announcement and based on the previous work on molybdate^35^, sulfate^36^, and phosphates^38^, exploration of other lithium-containing phosphates as cathodes led to the identification of olivine LiFePO_4_ as a cathode^39^ in 1997, ten years after the initial identification in 1987 of the polyanion class of cathodes and the inductive effect^35,36^.

The ability to increase the voltage drastically to as high as ~5 V in polyanion oxide cathodes^39,40^, for example, in LiMPO_4_ even with lower-valent couples like Co^2+/3+^ or Ni^2+/3+^ illustrates the power of the inductive effect imparted by the changes in metal-oxygen bonding in tuning the operating voltages. Accordingly, the polyanion oxide class with sulfates, phosphates, and silicates has become diverse compared to the first two classes of oxide cathodes (layered and spinel oxides) in terms of versatility and number of materials, not only for lithium-ion batteries, but also for sodium-ion batteries^40^. For example, polyanion oxides like Li_3_V_2_(PO_4_)_3_, Na_3_V_2_(PO_4_)_3_, and Na_3_V_2_(PO_4_)_2_F_3,_ and LiFePO_4_ have become appealing cathodes for lithium-ion or sodium-ion batteries^40–43^.

## Advantages and disadvantages of the oxide cathodes

The three classes of oxide cathodes discussed above have their advantages and disadvantages. Both the layered and spinel class of oxides offer good electronic conductivity, while the polyanion oxide class suffers from poor electronic conductivity. Therefore, the polyanion oxide cathodes require the particles to be synthesized small and coated with conductive carbon, which often increases the processing cost and introduces inconsistencies in performances. Both layered and spinel oxides have close-packed structure with high densities, while the polyanion class of oxides generally have lower densities, which is further reduced by the necessity to make them as small particles coated with carbon, leading to a lower volumetric energy density^44^. Thus, the polyanion cathodes are generally less attractive for applications that require high volumetric energy density, such as portable electronic devices and electric vehicles, than the layered oxide cathodes.

However, the polyanion class of cathodes offer an important advantage of high thermal stability and better safety than the layered and spinel oxide cathodes as the oxygen is tightly bound to P, S, or Si with strong covalent bonds^45^. Also, the polyanion cathodes with optimally small particles coated with carbon can sustain high charge–discharge rates due to good structural integrity, despite a lower volumetric energy density. Moreover, polyanion cathodes are known with abundant transition metals like Fe, unlike the layered and spinel oxides, offering sustainability advantages; therefore, they are appealing for grid storage of electricity produced from renewable energy sources like solar and wind.

Between the layered and spinel oxides, layered oxides are more appealing with a wide range of compositions than spinel oxides due to the inability to stabilize highly oxidized M^3+/4+^ states by conventional synthetic processes for the spinel oxides. In fact, the spinel cathodes are largely limited to LiMn_2_O_4_, but even that is plagued by Mn dissolution and the consequent poisoning of graphite anodes and capacity fade particularly at elevated temperatures. However, substituting a small amount of lithium (e.g., 5 atom %) for Mn helps to reduce the problems to some extent. On the other hand, although LiMn_1.5_Ni_0.5_O_4_ is appealing due to the high operating voltage of ~4.7 V and the consequent power capability, its practical viability is hampered by the lack of adequate electrolytes that can survive at such high operating voltages.

## Outlook

It is clear that among the three classes of oxide cathodes, layered oxides are the favorite candidates at least in the near term, considering their high gravimetric and volumetric energy densities. However, cost and sustainability are becoming critical as we move forward with large-scale deployment of lithium-ion batteries for electric vehicles and potentially for stationary storage^46^. Also, there is an appetite to increase the energy density beyond the current level to keep up with the advances in portable electronic devices and enhance the driving range of electric vehicles. Accordingly, concerted efforts are made to increase the cathode capacity and lower the cost. In this regard, lithium-rich layered oxides, such as Li_1.2_Mn_0.6_Ni_0.2_O_2_, that is rich in Mn and cobalt-free became appealing due to lower cost and capacities as high as 300 mA h g^–1^ during the past 15 years^47,48^. However, as discussed in the layered oxide section earlier, the layered to spinel transitions due to the low OSSE of Mn^3+^ and the consequent voltage decay during cycling as well as Mn dissolution and the consequent poisoning of the graphite anode have been a challenge to employ them as a practical cathode. Intrigued by the involvement of oxide ions in the redox process of lithium-rich layered oxides, cathodes based on anion redox have become recently appealing, at least from a basic science point of view^49^. However, the long-term cycle stability of such cathodes in full cells needs to be fully evaluated as we move forward to assess their practical viability.

**Fig. 5:**
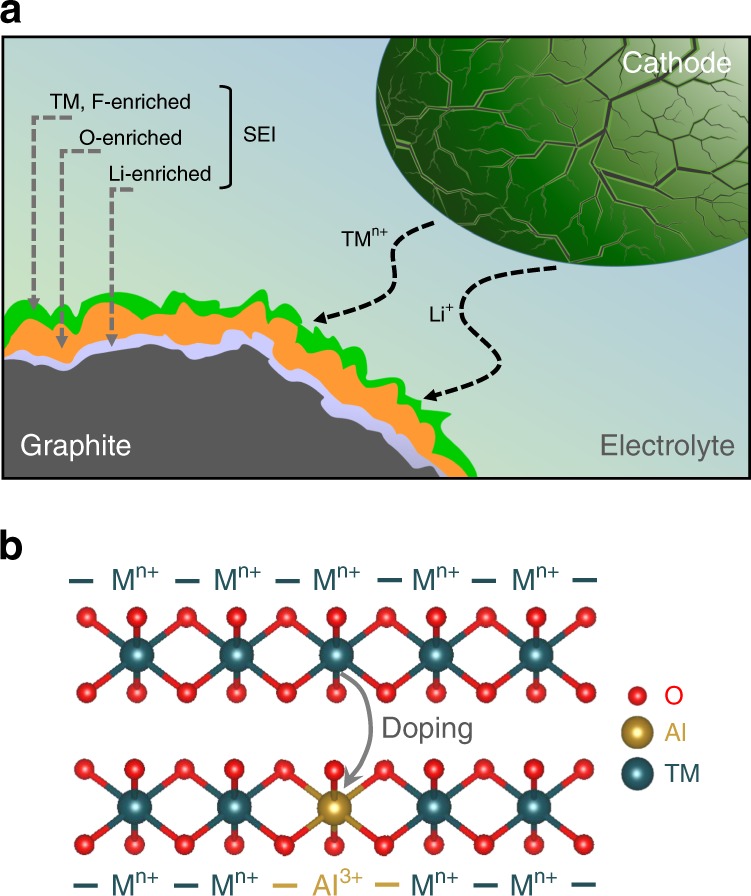
Challenges associated with high-nickel layered oxide cathodes and the role of cation doping. **a** Schematic illustration of the dissolution and migration of transition-metal ions from the cathode to the graphite anode and the consequent catalytic formation of thick SEI layers on the graphite anode. (**b**) Substitution of transition-metal ions with a small amount of inert ion like Al^3+^ that makes the lattice robust by perturbing the long-range metal-metal interaction and increasing the metal-oxygen bond strength and thereby suppressing metal-ion dissolution.

More recently, increasing the Ni content and lowering or eliminating the cobalt content in NMC cathodes is becoming much more prominent^50^. Intensive efforts are underway around the world with this strategy as Ni^2+^ or Ni^3+^ can be oxidized all the way to Ni^4+^ without encountering much oxygen loss from the lattice, unlike with Co, as discussed earlier in the layered oxide section. However, layered oxides with high Ni contents have three critical challenges: cycle instability, thermal instability, and air instability, all of which are related to the instability of Ni^3+^ or Ni^4+^ in contact with the liquid organic electrolyte or ambient air. This is understandable considering that only NiO with Ni^2+^ is known as a binary oxide and oxides like Ni_3_O_4_ or Ni_2_O_3_ with Ni^3+^ do not exist. Therefore, the chemically unstable Ni^4+^ ions that are generated on charging layered oxides with high Ni contents react aggressively with the electrolyte to form thick solid-electrolyte interphase (SEI) layers along with the dissolution of Ni and Mn, which then migrate to and deposit on the graphite anode and limit the cyclability^51,52^. The transition-metal deposition on graphite anodes catalyzes electrolyte decomposition and leads to the formation of a thick SEI layer with a multilayer structure as seen in Fig. 5, which increases with increasing number of cycles as more transition-metal ions dissolve and migrate to the anode. After a specific number of cycles, the SEI layer thickness also increases with increasing Ni content due to the increasing transition-metal dissolution and deposition on the graphite anode^53^. Furthermore, the phase transitions occurring in high-nickel cathodes at a high state-of-charge with volume changes introduce cracks with new surfaces on cycling, which further exaggerate the surface reactivity with the electrolyte and increase metal dissolution and SEI formation, resulting in rapid capacity fade as cycling progresses. This issue becomes much more prominent and serious particularly after large number of cycles, extending beyond, for example, 500 cycles^53^.

Intuitive bulk cation doping as well as surface stabilization strategies to minimize the volume changes, crack formation, and surface reactivity can help to overcome the challenges as we move forward^54^. For example, doping with a small amount of inert Al^3+^ for the transition-metal ions as seen in Fig. 5b increases electron localization by perturbing the long-range metal-metal interaction, decreases the long-range metal-oxygen covalence, makes the lattice robust with strong metal-oxygen bonds, and thereby suppresses transition-metal ion dissolution and improves the long-term cycle life. Such strategies also help to improve the thermal stability and air stability^55^. Overall, both controlled materials synthesis and advanced bulk and surface characterization methodologies can help to develop an in-depth basic science understanding and mitigate the issues as we move forward^51–55^.

The capacity of the three classes of insertion-reaction oxide cathodes discussed above are generally limited due to the limited number of crystallographic sites available as well as the large voltage steps encountered on going from one redox couple to another. For example, the voltage drops by more than 1 V on going from the V^4+/5+^ couple to the V^3+/4+^ couple, introducing challenges to employ them in practical applications. Ni is one unique candidate, which does not experience a voltage step on going through multiple redox couples, i.e., from the Ni^3+/4+^ couple to the Ni^2+/3+^ couple, as illustrated by the high-voltage LiMn_1.5_Ni_0.5_O_4_ spinel and layered NMC oxides. Considering the limitations in the capacity of insertion-reaction oxide cathodes, the alternative is to focus on conversion–reaction cathodes, such as sulfur and oxygen^56,57^. However, both lithium–sulfur and lithium–oxygen batteries face challenges, much more so with lithium–oxygen batteries than with lithium–sulfur batteries. Catalytic decomposition of electrolytes resulting in poor cycle life as well as sluggish reaction kinetics resulting in a large hysteresis between the charge and discharge voltages remain as daunting issues for lithium–oxygen batteries. On the other hand, enormous progress is being made with lithium-sulfur batteries in recent years, hopefully making them viable^58^. However, the necessary practical parameters and metrics need to be seriously considered and followed through to make the lithium-sulfur technology successful. In this regard, a target consisting of “five 5s” and employing such targets in pouch cells could help as we move forward with lithium-sulfur batteries^59^. The five targets are a sulfur loading of >5 mg cm^–2^, a carbon content of <5%, an electrolyte to sulfur (E/S) ratio of <5 µL mg^–1^, an electrolyte to capacity (E/C) ratio of <5 µL (mA h)^–1^, and a negative to positive (N/P) ratio of <5.

## Conclusion

In summary, concerted basic science research led to the identification of three classes of transition-metal oxide cathodes in the 1980s with much higher operating voltages than the previously explored sulfide cathodes for lithium-based batteries. They are layered oxides, spinel oxides, and polyanion oxides, and these three classes remain the viable practical cathodes and serve as a basis for future developments. The jump from sulfide cathodes to oxide cathodes was based on a simple idea that the top of the O^2–^:2p band lies at a lower energy than the top of the S^2–^:3p band, enabling access to lower-lying energy bands with higher oxidation states of transition-metal ions and a consequent increase in the operating voltage. The transition from simple oxide cathodes to polyanion oxide cathodes was based on the basic idea that a decrease in the covalency of the metal-oxygen bond by counter-cations (inductive effect) lowers the cathode redox energy and increases the operating voltage further compared to a simple oxide with the same redox couple. The higher operating voltages of oxide cathodes leading to higher energy densities and the presence of lithium in the as-synthesized cathodes prompted the commercialization of the modern-day lithium-ion batteries in the 1990s.

As we move forward with large-scale applications, there is demand to increase the energy density further while lowering the cost. In this regard, layered oxide cathodes with high nickel content have become appealing, but intuitive bulk and surface stabilization strategies are needed to overcome the cycle, thermal, and air instabilities associated with them and to realize their full potential. Alternatively, as the insertion-reaction oxide cathodes have a limitation in capacity due to the restricted number of available crystallographic sites for lithium insertion/extraction and encounter large voltage steps on going from one redox couple to another, there is intense research activity on conversion–reaction cathodes like sulfur and oxygen. However, they are hampered with serious challenges, but significant progress is being made with lithium-sulfur batteries. Nevertheless, critical target metrics in assembling the cells need to be vigorously pursued to assess the full potential of lithium-sulfur batteries. Finally, innovative synthesis and processing approaches along with advanced characterization methodologies and computational analysis could aid the discovery of new materials as we continue our journey to realize a cleaner, more sustainable planet.
